# Wild pigs as sentinels for hard ticks: A case study from south-central Florida

**DOI:** 10.1016/j.ijppaw.2018.04.003

**Published:** 2018-04-30

**Authors:** Mary M. Merrill, Raoul K. Boughton, Cynthia C. Lord, Katherine A. Sayler, Bethany Wight, Wesley M. Anderson, Samantha M. Wisely

**Affiliations:** a Department of Environmental and Global Health, College of Public Health and Health Professions, University of Florida, PO Box 100188, Gainesville, FL 32610, USA; b Department of Wildlife Ecology and Conservation, Institute of Food and Agricultural Sciences, University of Florida, 110 Newins-Ziegler Hall, Gainesville, FL 32611, USA; c Florida Medical Entomology Laboratory, Institute of Food and Agricultural Sciences, University of Florida, 200 9th St SE, Vero Beach, FL 32962, USA

**Keywords:** Ticks, Vector surveillance, *Sus scrofa*, Parasite-host ecology, Florida, Wild pigs

## Abstract

As a result of shifts in the habitable range of ticks due to climate change and the ongoing threat of exotic tick species introductions, efficient surveillance tools for these pests and disease vectors are needed. Wild pigs are habitat generalists, distributed throughout most of the United States, and often hunted recreationally or removed as part of management programs, making them potentially useful sentinel hosts for ticks. We compared ticks collected from captured wild pigs and standard tick dragging methods on a south-central Florida cattle ranch from May 2015–August 2017. Three hundred and sixteen wild pigs were surveyed, and 84 km spanning three habitat types (seminative pasture, improved pasture, and hammock) were dragged. In total, 1023 adults of four species (*Amblyomma auricularium*, *Amblyomma maculatum*, *Dermacentor variabilis*, and *Ixodes scapularis*) were collected from wild pigs, while 39 adults of three species (*A. auricularium*, *A. maculatum*, and *I. scapularis*) were collected from drags. Only one immature specimen, a nymph, was collected from a pig, while dragging collected 2808 larvae and 150 nymphs. *Amblyomma maculatum* comprised 96% of adults collected from pigs, while *A. maculatum*, *I. scapularis,* and *A. auricularium* comprised 38%, 33%, and 28% of adults collected from drags, respectively. Adults of all tick species found on drags were found on pigs, and wild pig surveillance detected adults of an additional species not found on drags. Dragging was far superior for collection of immatures but not for adults of most species found in this study. These findings suggest wild pigs could be used as a sentinel for the detection of tick species. When combined with ongoing wild pig research, hunting, or management, wild pig surveillance can provide an effective method to survey for adult tick presence of some species of interest and may assist in tracking the range expansion of some tick species.

## Highlights

• Surveillance for ticks, and potential disease risk in an area, is needed.
• Wild pigs are found throughout the US and may be useful sentinel hosts for ticks.
• We compared two methods for tick detection in south-central FL.
• Sampling from wild pigs detected adults of more tick species than dragging.
• Dragging was more successful than wild pig sampling at detecting immature ticks.

## Graphical abstract

**Figure:**
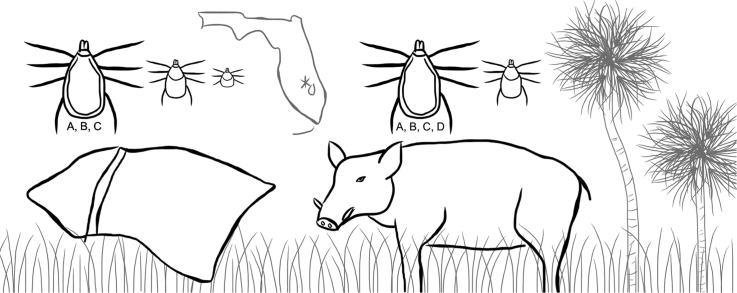

## 1 Introduction

The need for proactive and efficient methods of surveillance for ticks is increasing. Climate change causes shifts in the habitable range of vectors, allowing them to expand into new regions (Dantas-Torres, 2015). In addition, over the past few decades, at least 99 exotic tick species, including known vectors of disease, have been imported to the United States or discovered at ports of entry (Keirans and Durden, 2001). As a result of the changing climate and increased trade of domestic livestock, ticks and their associated pathogens are emerging in new locations and threatening the health of humans and animals (Barré and Uilenberg, 2010). Early detection of tick range expansions and of exotic tick species introductions is critical to inform veterinary and public health response measures.

The goals of tick surveillance vary, but often include monitoring for the emergence of exotic species or assessing range, habitat use, and host use for native tick species. Methods of tick surveillance include both environmental or host surveys (Estrada-Pena et al., 2013). Environmental surveys for host-seeking ticks are wide-ranging and include cloth dragging and flagging, walking surveys, surveys of animal nests, and carbon-dioxide-baited or other attractant-baited traps (Koch and McNew, 1981; Schulze et al., 1986, 1997; Ginsberg and Ewing, 1989; Petry et al., 2010; Cohnstaedt et al., 2012; Portugal and Goddard, 2015; Mays et al., 2016). Host sampling includes surveys of humans, companion animals, domestic livestock, and wild animals trapped for research or management or harvested by hunters (Ogden et al., 2006; Rand et al., 2007; Hamer et al., 2009; Cohnstaedt et al., 2012; Hertz et al., 2017; Mertins et al., 2017).

The efficacy of all surveillance types may vary depending on tick biology, tick life stage, tick host-seeking methods, host selection, habitat type, and weather (Ginsberg and Ewing, 1989; Wilson, 1994; Schulze et al., 1997; Petry et al., 2010; Cohnstaedt et al., 2012). Drag method results are highly influenced by habitat type and vegetation structure, and even within habitat types, ticks are often heterogeneously distributed (Wilson et al., 1988; Dobson et al., 2011). For example, if ground vegetation prevents a drag-cloth from reaching the lower levels of vegetation or leaf litter, this may prevent collection of ticks which quest at low heights. Additionally, host-seeking tick surveillance methods are affected by both time of day and short-term environmental conditions (Wilson, 1994). In contrast, host surveillance is not as affected by vegetation structure or short-term weather variables (Wilson, 1994; Estrada-Pena et al., 2013), and sentinel animals are available to host-seeking ticks for longer periods of time than standard drag sampling. Sampling of sentinel animals may better detect ticks that are at low densities in the environment or not responsive to host-seeking tick surveillance, and has been shown to provide informative assessments of tick control efforts (Ginsberg and Ewing, 1989; Schulze et al., 1997; Hamer et al., 2009; Polito et al., 2013).

Good sentinel hosts are species which are readily observable and more likely than others to be exposed to ticks (Halliday et al., 2007). The ideal sentinel host depends on the tick species and life stage of interest. Tick attraction to and ability to utilize a sentinel host are necessary factors for any sentinel tick surveillance. In the case of detection of adults of many tick species, an ideal sentinel would be a vertebrate host that has a medium to large body size (Esser et al., 2016), is regularly handled in large numbers, and utilizes diverse habitats over a large but relatively stable home range. Surveys of domestic animals such as dogs (*Canis familiaris* L.) and cattle (*Bos taurus* L., *Bos indicus* L., and their crosses) are often utilized to assess tick distribution, tick-borne disease risk, and tick control methods as they fit many of these criteria (Barnard, 1981; Johnson et al., 2004; Hamer et al., 2009; Polito et al., 2013; Pompo et al., 2016). However, differing vector control practices, such as the use of acaricides, complicate comparability of domestic animal surveys, may protect animals from attaching ticks, and interfere with the aim of tick species detection (Hamer et al., 2009; Pompo et al., 2016). Large-bodied wildlife, particularly game or pest species which are harvested regularly, can provide a useful alternative. Examination of white-tailed deer (*Odocoileus virginianus* Zimmermann) and other game at hunter-check stations has proven valuable for assessing tick distribution over large areas and understanding the role large-bodied wildlife play in the ecology of ticks (Allan et al., 2001; Cortinas and Kitron, 2006; Yabsley et al., 2009; Hertz et al., 2017).

Wild pigs (*Sus scrofa* L.) are a large-bodied, non-native, invasive mammal introduced to the mainland United States by European explorers in the 16th century, with multiple reintroductions occurring since (Mayer and Brisbin, 1991). Wild pigs consist of released or escaped domestic swine, Eurasian wild boar, and their hybrids. Over the past few decades, the distribution of wild pigs in the United States has expanded dramatically (Gipson et al., 1998; Bevins et al., 2014). Wild pigs have now been reported in most states, and share space and resources with other wildlife, domestic livestock, and humans. Their wide geographical range and ability to thrive in multiple habitat types, combined with ongoing and widespread removal efforts as well as recreational hunting across the United States, suggest that wild pigs are a potentially useful and easily accessible sentinel species.

Wild pigs in the United States typically have home ranges of multiple square kilometers (Kurz and Marchinton, 1972; Adkins and Harveson, 2007; Mersinger and Silvy, 2007; Friebel and Jodice, 2009) and utilize a variety of habitats (Wood and Brenneman, 1980; Singer et al., 1981; Barrett, 1982; Baber and Coblentz, 1986). Wild pigs have previously been found to host multiple native and non-native tick species with differing habitat preferences, including important pests of wildlife and many well-known vectors of livestock and human disease (Table 1). Surveillance of wild pigs detected the geographic expansion of *Dermacentor variabilis* in Texas (Sanders et al., 2013). However, unlike other sympatric wildlife, wild pigs were not found to be important hosts of the economically important cattle fever ticks (*Rhipicephalus (Boophilus) annulatus* (Say) and *Rhipicephalus (Boophilus) microplus* (Canestrini)) near the Mexico/Texas border (Corn et al., 2016). Currently, information is lacking on how active tick surveillance using wild pigs in the United States compares to dragging methods. Surveillance of wild pigs may provide a way to sample greater areas in environments that are not conducive to drag methods, to detect certain species which do not respond to dragging, and to detect non-native tick species before they are at numbers sufficient to detect through drags.

**Table 1:** Review of tick species collected from wild pigs in the United States.

Species	Location	Reference
*Amblyomma americanum*	Alabama	Smith et al., 1982
	Arkansas	Smith et al., 1982
	Florida	Greiner et al., 1984; Allan et al., 2001; Hertz et al., 2017
	Georgia	Hanson and Karstad, 1959; Smith et al., 1982
	Kentucky	Fritzen et al., 2011
	South Carolina	Smith et al., 1982
	Texas	Shender et al., 2002; Sanders et al., 2013
	Virginia	Smith et al., 1982
*Amblyomma auriculariu m*^a^	Florida	Allan et al., 2001; Mertins et al., 2017
*Amblyomma breviscutatum*	Guam	Cleveland et al., 2017
*Amblyomma cajennense*^b^	Texas	Coombs and Springer, 1974; Shender et al., 2002; Sanders et al., 2013
*Amblyomma maculatum*	Arkansas	Smith et al., 1982
	Florida	Smith et al., 1982; Greiner et al., 1984; Allan et al., 2001; Hertz et al., 2017
	Georgia	Hanson and Karstad, 1959
	Mississippi	Smith et al., 1982
	Texas	Coombs and Springer, 1974; Shender et al., 2002; Sanders et al., 2013; Corn et al., 2016
*Amblyomma mixtum*	Texas	Corn et al., 2016
*Amblyomma tenellum*	Texas	Corn et al., 2016
*Dermacentor albipictus*	Texas	Sanders et al., 2013
	New Hampshire	Musante et al., 2014
*Dermacentor halli*	Texas	Sanders et al., 2013; Corn et al., 2016
*Dermacentor variabilis*	Florida	Smith et al., 1982; Greiner et al., 1984; SCWDS records reported in Davidson et al. (1987); Allan et al., 2001; Hertz et al., 2017
	Georgia	Hanson and Karstad, 1959; Smith et al., 1982
	Kentucky	Fritzen et al., 2011
	South Carolina	Smith et al., 1982
	Tennessee	Henry and Conley, 1970
	Texas	Springer, 1973; Shender et al., 2002; Sanders et al., 2013; Corn et al., 2016
*Ixodes scapularis*	Florida	Smith et al., 1982; Greiner et al., 1984; SCWDS records reported in Forrester (1992); Allan et al., 2001; Hertz et al., 2017
	Georgia	Smith et al., 1982
	Louisiana	Smith et al., 1982
	South Carolina	Smith et al., 1982
	Texas	Coombs and Springer, 1974; Sanders et al., 2013

a Non-native to the United States.

b Based on available information at the time, specimens were originally identified by the authors as *A. cajennense*; however, in 2014, *A. cajennense* was confirmed to be a complex of six species (Nava et al., 2014), with previously identified *A. cajennense* from Texas likely representing the resurrected *A. mixtum*.

The objectives of this study were to compare the ability of cloth dragging and wild pig sampling to detect the presence, abundance, and life stages of tick species on a working beef cattle ranch in south-central Florida. We expected that wild pig samples would predominantly capture adults, as suggested by previous studies (Greiner et al., 1984; Hertz et al., 2017). Immature stages of many tick species found in south-central Florida, such as *Amblyomma maculatum* Koch, *Dermacentor variabilis* (Say), and *Ixodes scapularis* Say, commonly parasitize small and medium vertebrate hosts (Bishopp and Trembley, 1945; Clymer et al., 1970; Keirans et al., 1996; Kollars et al., 2000; Teel et al., 2010). Thus, we expected that drag sampling would produce higher numbers of immatures than sampling wild pigs. We hypothesized that sampling wild pigs would detect greater numbers and higher species richness of adults than dragging since wild pigs spend time in multiple, diverse microhabitats suitable for different tick species.

## 2 Materials and methods

### 2.1 Study site

**Fig. 1:**
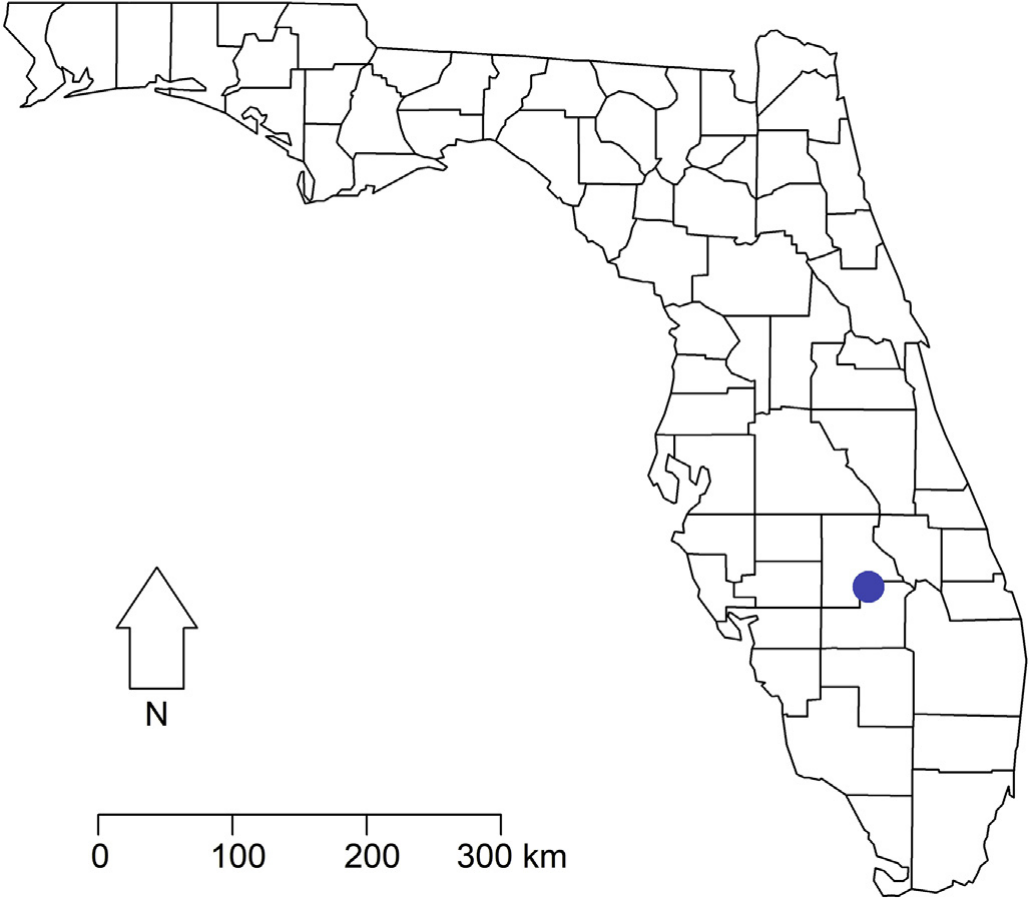
Location of Buck Island Ranch, Lake Placid, Florida denoted by blue circle. (For interpretation of the references to color in this figure legend, the reader is referred to the Web version of this article.)

The MacArthur Agro-ecology Research Center, a division of Archbold Biological Station, is located at Buck Island Ranch in Lake Placid, Florida (Fig. 1) (Swain et al., 2013). At the site, around 3000 cattle utilize two pasture types referred to as “improved” and “seminative.” In the mid-1900s, ranch owners plowed and planted most of the upland dry prairie portions of the ranch with exotic forage species such as Bahia grass (*Paspalum notatum*), as well as installed a well-developed system of ditches for water regulation, creating improved pastures. Seminative pastures are at lower elevations than the improved pastures and still host many native wet prairie plant species. Multiple stands of trees, regionally referred to as “hammocks,” are found on the ranch. These hammocks are closed canopy forests with moist soil, typically dominated by evergreen species such as live oak (*Quercus virginiana*) and cabbage palm (*Sabal palmetto*), with a fairly open shrub layer and sparse herb layer (U.S. Fish and Wildlife Service, 1999). Buck Island Ranch also contains two large wetland sites which together total more than 700 acres, and hundreds of smaller seasonal wetlands which are typically less than 1.5 acres in size (Swain et al., 2013; MacArthur Agro-ecology Research Center, 2014). The ranch hosts many native wildlife species such as white-tailed deer, wild turkey (*Meleagris gallopavo* L.), and Northern bobwhite (*Colinus virginianus* L.), as well as invasive species such as wild pigs (MacArthur Agro-ecology Research Center, 2014).

### 2.2 Host-seeking tick surveillance

Host-seeking tick surveillance was conducted from May 14, 2015 to August 29, 2017 by dragging a white, 1 m^2^ corduroy or velveteen cloth along the ground and over vegetation in three habitat types: improved pastures, seminative pastures, and hammocks for up to 1000 m per drag. Tick dragging was performed during daylight hours when no dew was present on the ground. The cloth was checked for ticks every 10 m, and any ticks found were collected, kept alive on ice packs or at ambient temperature, and later the same day stored in 90% ethanol, frozen at −20 °C, or both. For each drag, data such as start and end time, global positioning system coordinates for the beginning and end of each transect, total drag distance, habitat type, and pasture name were recorded. Monthly drags were conducted in each of the three habitat types. For the first four months of the study (May–August 2015), we sampled from 100 to 300 m per drag at adventitious sites in each of the habitats. These methods detected extremely few ticks of any life stage, so for the remainder of the study we increased our target drag distance at each site to 1000 m. Additionally, when possible, we conducted drags in pastures where ranch personnel and research staff informally relayed finding ticks on themselves or cattle or where we had successfully collected ticks previously. Incorporating this input risked the potential for artificial inflation of detected densities of host-seeking ticks. However, based on our limited data from the first four months, we believed incorporation of local knowledge was a necessary and reasonable component of a host-seeking tick surveillance plan.

### 2.3 Tick collection from animals

Wild pigs were sampled from May 22, 2015 to May 09, 2017. Trapping of live wild pigs was conducted for a broader study of their movement and ecology, and we opportunistically collected ticks from wild pigs trapped for those purposes. Animal handling was approved by University of Florida Institutional Animal Care and Use Committee #201408495. Wild pigs were trapped in large corral or box-style traps baited with fermented corn. Traps were placed in areas of suspected high wild pig activity evidenced by direct sightings or other indicators such as fresh wild pig tracks, rooting, or droppings. Traps were placed in shaded areas, often within or along the edge of hammocks to reduce the potential for heat stress on the animals. All traps were set in the evening and checked early the following morning, at which point any captured wild pigs were guided through a squeeze-chute or chemically immobilized following appropriate procedures (Kreeger and Arnemo, 2012). When sample collection was completed on anesthetized individuals, reversal drugs were administered, and animals were released at the point of capture. If the animals were part of a removal effort, they were transported by a State Veterinarian's Office registered Feral Swine Dealer to an approved abattoir (Florida Department of Agriculture and Consumer Services, accessed December 04, 2017). Additional hunter harvested wild pigs were sampled when available. The ears of wild pigs were thoroughly checked for ticks both visually and by feeling the surface of the skin. Ticks were removed from wild pigs using clean, fine-tipped forceps or other available removal tools, kept alive on ice packs or at ambient temperature, and later the same day stored in 90% ethanol, frozen at −20 °C, or both.

### 2.4 Species identification

Because wild pigs in this region are primarily hosts for adult ticks (Greiner et al., 1984) and morphological keys are limited for the immatures of many exotic species, we identified adults only. Adults were identified to species morphologically using taxonomic keys (Keirans and Litwak, 1989; Guzman-Cornejo et al., 2011). Representative specimens were deposited in the U.S. National Tick Collection, Georgia Southern University, Statesboro, Georgia.

### 2.5 Statistical analysis

Host-seeking tick density was calculated for each drag as the average number of ticks collected per 10 m^2^. To determine associations between habitat and life-stage for host-seeking ticks, we used a Poisson regression model for count data. We included distance dragged as an offset in the model to adjust for the differences in sampling size among habitats. For wild pigs, prevalence of infestation was defined as the proportion of wild pigs infested by a tick species among all wild pigs examined during the specified time period (monthly or throughout the entire study). Mean intensity of infestation was defined as the number of individuals of a tick species collected divided by the total number of wild pigs infested by that species during the specified time period (Rózsa et al., 2000). Mean abundance of ticks was calculated as the total number of ticks collected divided by the total number of pigs examined during the specified time period. Confidence intervals for the prevalence, abundance, and intensity of tick infestation on wild pigs were calculated using the non-parametric bootstrap with 2000 replicates (Davison and Hinkley, 1997; Canty and Ripley, 2016). Data were analyzed in R version 3.3.2 (R Core Team, 2016).

## 3 Results

The total number, species, and life stages of ticks collected from drags and wild pigs are presented in Table 2. *Amblyomma maculatum* was the most commonly collected tick from both wild pigs and drags, accounting for 96% and 38% of total adults collected, respectively. Thirty-nine adults of three ixodid tick species (*Amblyomma auricularium* (Conil), *A. maculatum*, and *I. scapularis*) were collected from 83,916 square meters of drags. One thousand and twenty-three adults of four ixodid tick species (*A. auricularium*, *A. maculatum*, *D. variabilis*, and *I. scapularis*) were collected from 316 wild pigs. One hundred and fifty nymphs and 2808 larvae were collected from drags. Only one immature specimen, a nymph, was collected from a wild pig during this study.

**Table 2:** Ticks collected by drag-sampling from May 14, 2015 to August 29, 2017 and from wild pigs from May 22, 2015 to May 09, 2017 at Buck Island Ranch, Lake Placid, Florida.

Habitat	Cumulative drag distance (km)	Larvae	Nymphs	Adults	AMAU^a^		AMMA^a^		DEVA^a^		IXSC^a^		Unidentified^a^^,^^b^
					M	F	M	F	M	F	M	F	
Hammock	18.3	2794	138	21	6	5	0	0	0	0	6	4	0
Improved	30.8	1	8	4	0	0	1	3	0	0	0	0	0
Seminative	34.9	13	4	14	0	0	4	7	0	0	3	0	0
Total	84.0	2808	150	39	6	5	5	10	0	0	9	4	0

Host	Total sampled												
Wild pigs	316	0	1	1023	3	8	653	326	2	10	0	8	12

a Species information included only for adults.

b Unidentified specimens damaged beyond identification either on the host or during removal; AMAU = *A. auricularium*, AMMA = *A. maculatum*, DEVA = *D. variabilis*, IXSC = *I. scapularis*.

**Fig. 2:**
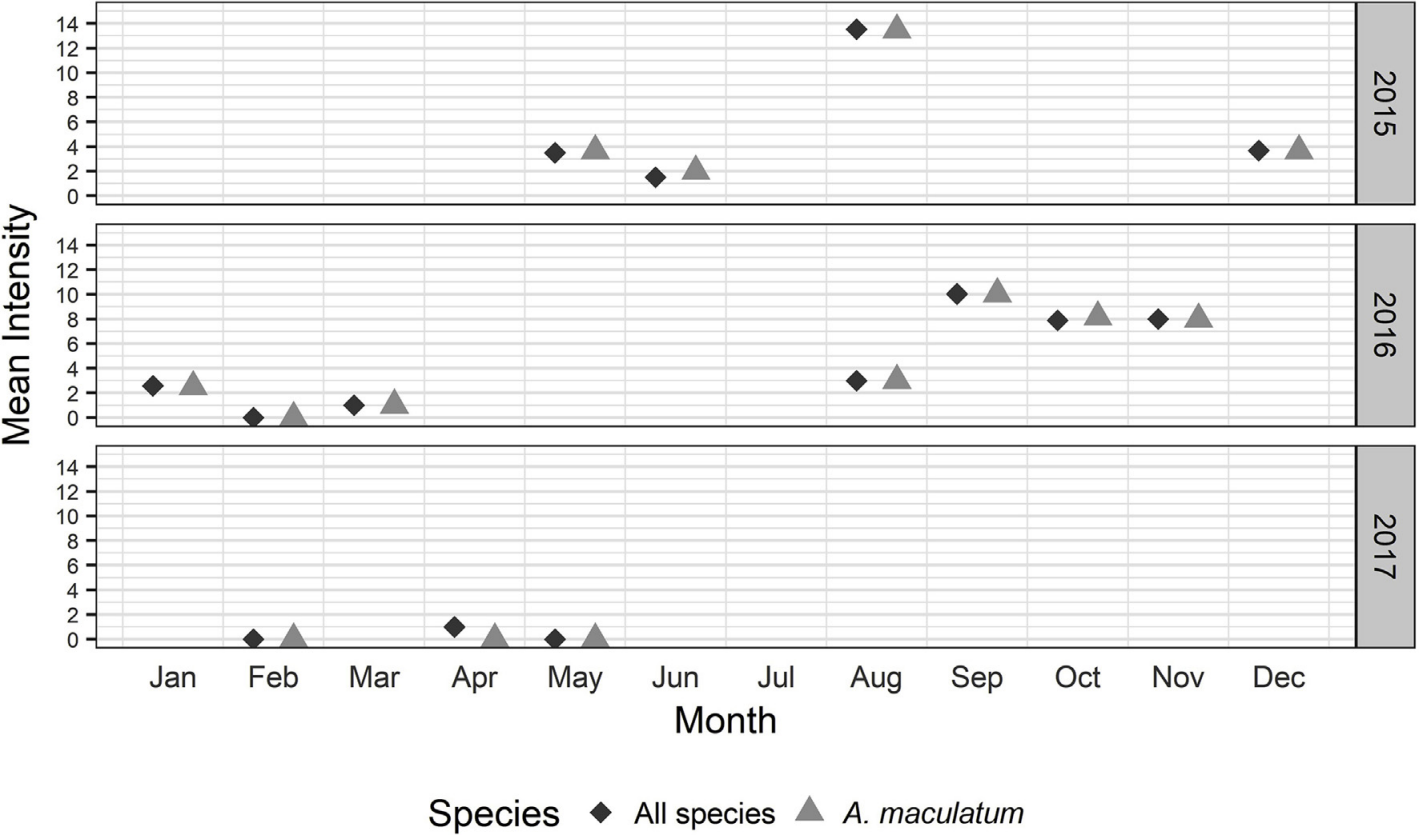
Mean intensity of infestation of adult ticks collected from wild pigs from May 22, 2015 to May 09, 2017. Ticks which could not be identified to species were excluded from this figure. Values of zero indicate that wild pigs were sampled during that month, but no adults of the indicated species were collected.

**Fig. 3:**
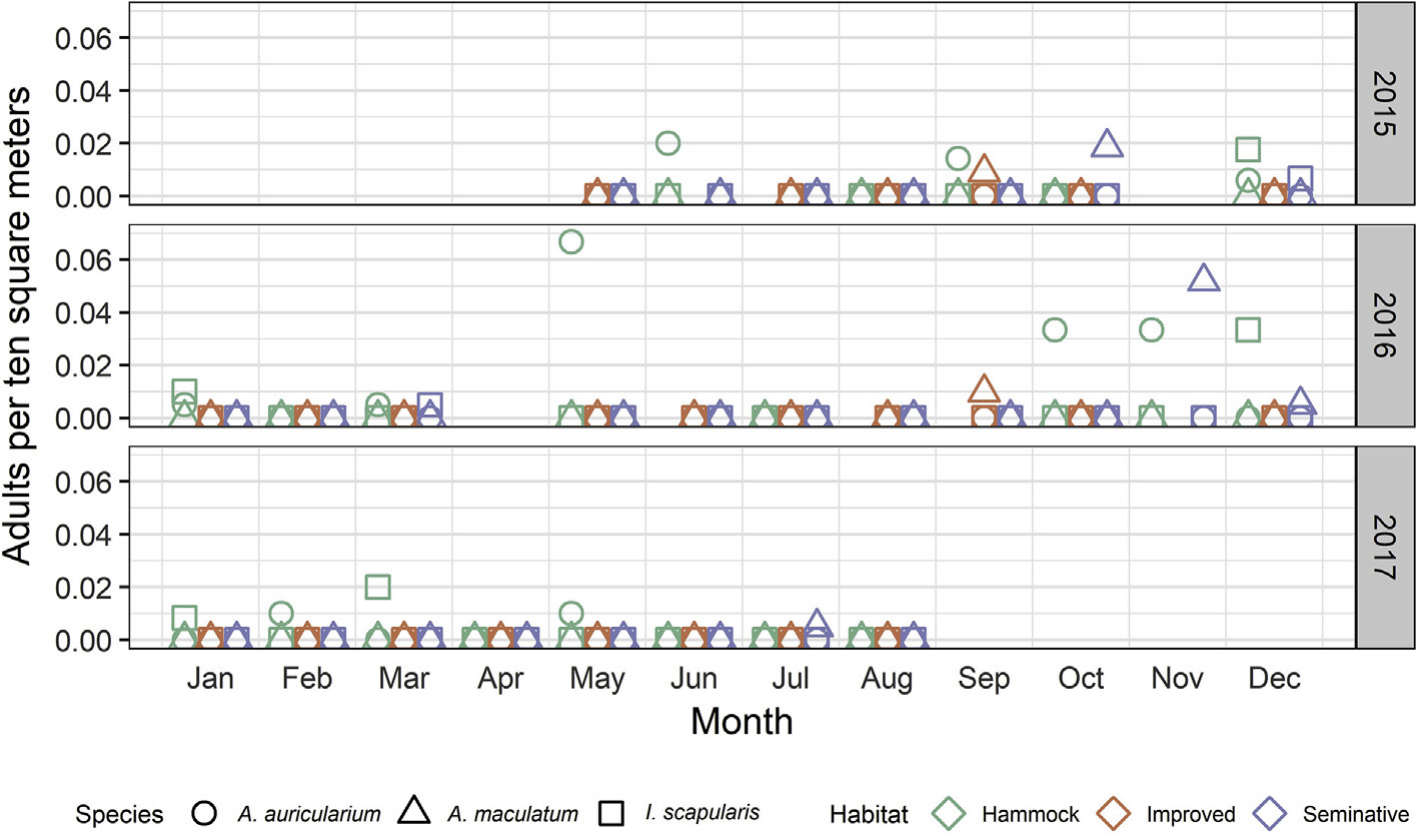
Average density of adults collected by dragging from May 14, 2015 to August 29, 2017. Values of zero indicate that drags were conducted during that month in the specified habitat, but no adults of the indicated species were collected. Symbol colors denote habitat and symbol shapes denote tick species. (For interpretation of the references to color in this figure legend, the reader is referred to the Web version of this article.)

Wild pig sampling was conducted during fourteen of the total 28 months of this study. Drag sampling in at least two of the three habitat types was successfully conducted 26 of the total 28 months of this study, and drag sampling was conducted in all three habitats in nineteen of 28 months. Gaps in drag sampling were due to poor weather conditions (rain or extreme wind) or flooding of pastures during scheduled study site visits. Fig. 2, Fig. 3 display the specific months during which wild pig sampling and drag sampling were successfully conducted, respectively.

**Fig. 4:**
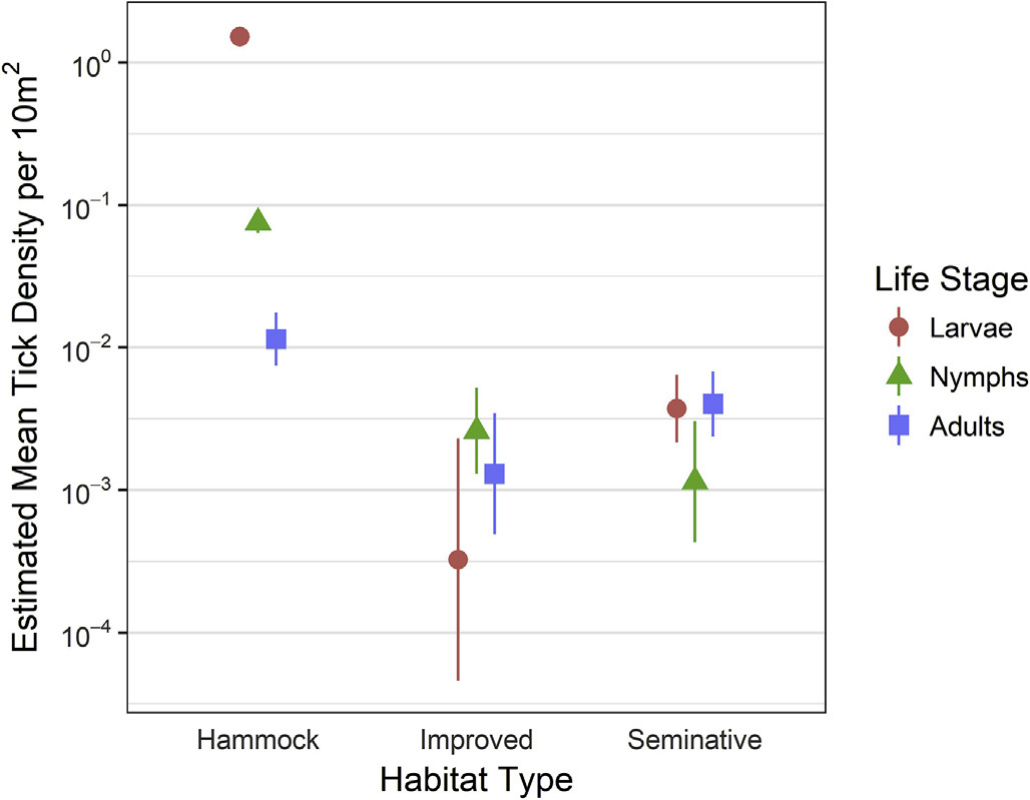
Estimated mean density of host-seeking ticks per 10 m^2^ by life stage and habitat type with 95% confidence intervals shown as vertical bars. Numerical values for the estimated mean densities and 95% confidence intervals are reported in Table S5.

Hammock was the most productive habitat for collecting host-seeking ticks of all life stages, particularly immatures. The estimated mean densities of host-seeking ticks for each life stage and habitat are displayed in Fig. 4. Habitat type, life stage, and the interaction of habitat type and life stage all had significant effects on the estimated mean density of host-seeking ticks (Table S1). Due to the low number of adults collected on drags (≤15 of any species), we did not conduct statistical analysis of habitat associations for different species of adults. However, from drag sampling, adults of *A. auricularium* were found only in hammock habitat, *A. maculatum* were found in both seminative and improved pasture habitat, and *I. scapularis* were found in both hammock and seminative pasture habitat, although the majority (10/13) were found in hammock habitat.

The prevalence, abundance, and intensity of infestation of tick species on wild pigs are recorded in Table 3, and the monthly mean intensity of infestation is displayed in Fig. 2. Overall, 40 percent of 316 wild pigs were infested with *A. maculatum*. Three percent of wild pigs were infested with *A. auricularium* and *D. variabilis*, and less than two percent were infested with *I. scapularis*. *Amblyomma maculatum* was both more prevalent and more abundant than all other tick species (Table 3). *Amblyomma maculatum* was collected from pigs almost every month sampled, with the exception of February 2016 and 2017, and April and May of 2017. *Amblyomma maculatum* had the highest intensity of infestation on wild pigs every month, with the exceptions of April 2017 and months where no ticks were collected (Fig. 2, Table 3). The 95% confidence intervals for prevalence, abundance, and intensity are included in Tables S2–S4.

**Table 3:** Prevalence, abundance, and intensity of tick infestation of wild pigs from May 22, 2015 to May 09, 2017 at Buck Island Ranch, Lake Placid, Florida. The 95% confidence intervals for prevalence, abundance, and intensity are included in Supplementary Tables 2–4.

2015	Pigs sampled	Prevalence				Abundance				Intensity			
		AMAU	AMMA	DEVA	IXSC	AMAU	AMMA	DEVA	IXSC	AMAU	AMMA	DEVA	IXSC
May	7	0.0	42.9	28.6	0.0	0.00	1.57	0.43	0.00	–	3.67	1.50	–
June	10	0.0	20.0	20.0	0.0	0.00	0.40	0.20	0.00	–	2.00	1.00	–
August	14	0.0	78.6	7.1	0.0	0.00	10.57	0.07	0.00	–	13.45	1.00	–
December	34	0.0	58.8	5.9	11.8	0.00	2.15	0.06	0.18	–	3.65	1.00	1.50
2016													
January	11	0.0	54.5	9.1	9.1	0.00	1.36	0.18	0.09	–	2.50	2.00	1.00
February	4	0.0	0.0	0.0	0.0	0.00	0.00	0.00	0.00	–	–	–	–
March	26	0.0	3.8	0.0	0.0	0.00	0.04	0.00	0.00	–	1.00	–	–
August	2	0.0	50.0	0.0	0.0	0.00	1.50	0.00	0.00	–	3.00	–	–
September	26	0.0	96.2	0.0	0.0	0.00	9.65	0.00	0.00	–	10.04	–	–
October	81	7.4	70.4	1.2	1.2	0.09	5.74	0.01	0.01	1.17	8.16	1.00	1.00
November	1	0.0	100.0	0.0	0.0	0.00	8.00	0.00	0.00	–	8.00	–	–
2017													
February	7	0.0	0.0	0.0	0.0	0.00	0.00	0.00	0.00	–	–	–	–
April	85	4.7	0.0	1.2	0.0	0.05	0.00	0.01	0.00	1.00	–	1.00	–
May	8	0.0	0.0	0.0	0.0	0.00	0.00	0.00	0.00	–	–	–	–
**Total Study**	**316**	**3.2**	**40.2**	**3.2**	**1.9**	**0.03**	**3.10**	**0.04**	**0.03**	**1.10**	**7.71**	**1.20**	**1.33**

Calculations based on adults identified to species. Prevalence calculated as the number of pigs infested divided by the number of pigs surveyed and expressed as a percentage. Abundance calculated as the sum of ticks collected divided by the number of pigs surveyed. Intensity calculated as the sum of ticks collected divided by the number of pigs infested. AMAU = *A. auricularium*, AMMA = *A. maculatum*, DEVA = *D. variabilis*, IXSC = *I. scapularis*.

Wild pig sampling detected a greater richness of adults than dragging: four species versus three, respectively. The relative number of adults detected by each sampling method varied by species. Wild pig sampling detected more than 65 times the total number of *A. maculatum* adults detected by dragging. Additionally, wild pig surveillance detected adults of one species, *D. variabilis*, that was not detected from drags. However, wild pig and drag sampling detected similar numbers of *A. auricularium* and *I. scapularis*, though male *I. scapularis* were found only on drags, not on wild pigs.

## 4 Discussion

We found wild pigs to be suitable sentinels for detection of adults of the four tick species collected in this survey of a south-central Florida cattle ranch. In this study, all wild pigs examined were either part of an already ongoing research project or harvested by recreational hunters. Thus, convenience sampling of ticks from wild pigs in cooperation with public and private partners can provide valuable insight into the presence of certain tick species that may not be detectable through drag-sampling, either due to low densities in the environment or limited response to drag methods. The ability of wild pigs to move large distances through multiple habitat types over the period of a few days is both a strength and weakness of wild pig surveillance. This allows wild pigs to contact multiple tick species with differing habitat preferences, potentially resulting in greater species richness; however, it does not allow for insights into tick habitat associations. For example, in our study, wild pig surveillance detected a greater species richness of adults than drag surveys, but drag surveys provided some information on the habitat associations of ticks. Dragging was also more productive than wild pig sampling for collecting immature life stages of ticks, which can be important for informing human disease risk (Piesman et al., 1987; Barbour and Fish, 1993).

Gulf Coast ticks (*A. maculatum*) were the species most commonly collected from wild pigs and the most commonly collected adults from drags. Wild pig surveillance detected more than 65 times the number of adult *A. maculatum* than drags. We found a prevalence of *A. maculatum* infestation on wild pigs of 40 percent, a mean intensity of nearly 8 ticks per infested pig, and a mean abundance of three ticks per pig. Of the months sampled, the mean intensity of *A. maculatum* on wild pigs was greatest from August–November (Fig. 2). We detected adult Gulf Coast ticks through drags in both seminative and improved pastures, but not in hammocks. The Gulf Coast tick is found throughout much of the Western Hemisphere. In the United States, the established population is mainly distributed throughout the Gulf and Atlantic Coastal states of the southeast, but recently widespread distribution and established populations have been reported in more northern states (Florin et al., 2014; Trout et al., 2010), with incidental reports as far north as Maine (Teel et al., 2010). Additionally, an isolated and expanding inland population is now established in Oklahoma and Kansas due to transportation of infested cattle (Teel et al., 2010). In Florida, the population density of Gulf Coast ticks is greater in the southern than northern regions (Allan et al., 2001; Hertz et al., 2017).

Prevalence of *A. maculatum* on wild pigs in Florida varies widely. Prevalences of infestation from 5 to 86% have been reported (Greiner et al., 1984; Allan et al., 2001; Hertz et al., 2017), with higher prevalences typically found in the southern region of the state. Our detected prevalence of 40% was less than half that detected in Glades County (the county bordering our study site to the south) from 1979 to 1981, where an 86% prevalence was detected (Greiner et al., 1984). Our focus on only the ears of wild pigs during surveys is not likely to explain our lower prevalence rate, as this is one of the primary attachment sites for *A. maculatum* (Teel et al., 2010), and Greiner et al. (1984) collected the vast majority of their *A. maculatum* specimens from the ears as well. Ungulates and carnivores account for the majority of reported hosts for adult *A. maculatum* (Teel et al., 2010). Cattle, white-tailed deer, horses (*Equus caballus* L.), coyotes (*Canis latrans* Say), and other suitable hosts are present at our study site, potentially reducing the burden of *A. maculatum* on wild pigs as well as potentially reducing the host-seeking population of *A. maculatum*, contributing to the low number collected on drags. We did not include a variable to account for “time since last presence of cattle” in our drag-sampling design. Cattle are major hosts of adult *A. maculatum*, and are known to host multiple other species (Teel et al., 2010; Pompo et al., 2016), and we did observe ticks on cattle at this site during the study period (M. M. Merrill, unpublished data, 2017). The presence of cattle in pastures and the human-driven movement of cattle among pastures may have both a short-term and long-term influence on the host-seeking tick population detected, and should be considered in future studies.

Wild pig sampling and drag sampling detected similar numbers of *A. auricularium*. April, July, and August were the only months during which we did not detect adult *A. auricularium* from drag samples at least one of the years sampled, and 2017 was the only year we drag-sampled in April (Fig. 3). The absence of detection during certain months likely reflects the low density at this study site rather than actual fluctuations in host-seeking tick abundance or behavior. A multi-year survey of vertebrates detected adult *A. auricularium* at relatively low and stable populations throughout the year in South Florida (Mertins et al., 2017). *Amblyomma auricularium* is not native to the United States, but is established in South Florida and has been previously reported from our study site in Highlands County (Merrill et al., 2016). Host-collected *A. auricularium* have been reported from multiple habitat types, including grass habitats, across the neotropical region and into the nearctic with no apparent habitat preference (Guglielmone et al., 2003). We found host-seeking adult *A. auricularium* exclusively in hammock habitat, even though our combined pasture sampling efforts (30.8 km in improved pastures, 34.9 km in seminative pastures) were more than three times the sampling effort in hammocks (18.3 km). In Florida, common hosts for *A. auricularium* include the nine-banded armadillo (*Dasypus novemcinctus* L.) and Virginia opossum (*Didelphis virginiana* Kerr) (Mertins et al., 2017), both of which are present at our study site. This tick has been reported on a wide range of host families and species, and wild pigs may serve as marginally important hosts (Allan et al., 2001; Mertins et al., 2017). Our overall prevalence of infestation of *A. auricularium* on wild pigs (3.2%) was slightly lower than that found on wild pigs from 2004 to 2007 in counties with known *A. auricularium* presence (8.4%) (Mertins et al., 2017).

Wild pig sampling and drag sampling produced similar numbers of *I. scapularis*, though in contrast to dragging, wild pig sampling did not detect male *I. scapularis*. We found a total black-legged tick (*Ixodes scapularis*) infestation prevalence of 1.9% on wild pigs. Our wild pig survey results were consistent with the prevalence (1%) found on wild pigs at nearby Fisheating Creek, Florida from 1979 to 1981 (Greiner et al., 1984), but much lower than the prevalence detected in central and south Florida (69.7%) and in the north and central regions of the state more recently (35%) (Allan et al., 2001; Hertz et al., 2017). *Ixodes scapularis* is established throughout most of Florida, and has been reported from a wide variety of hosts (Keirans et al., 1996; Eisen et al., 2016).

We collected twelve adult American dog ticks (*D. variabilis*) throughout the entire study, all from wild pigs. The 3% prevalence of infestation detected in this study was notably lower than that found in previous studies of wild pigs in Florida, for example 98% prevalence at Fisheating Creek (Greiner et al., 1984), and 56.9% from Central and South Florida (Allan et al., 2001). *Dermacentor variabilis* is distributed throughout the United States except parts of the Rocky Mountain region (Goddard, 1989; Dergousoff et al., 2013; James et al., 2015) and has been reported from the majority of Florida counties (James et al., 2015). As *D. variabilis* is frequently collected by drag sampling elsewhere (Garvie et al., 1978; Burg, 2001), it is likely the low number of *D. variabilis* collected from wild pigs combined with the lack of detection of *D. variabilis* adults from drag-sampling is due to low abundance of *D. variabilis* at our study site. Thus, surveillance of wild pigs proved a useful tool for detection of adults of this species.

We detected no *Amblyomma americanum* (L.) adults from wild pigs or drags. Though *A. americanum* is regularly collected from wild pigs and other wildlife in northern Florida, this tick is rarely collected from wildlife in the southern areas of the state (Greiner et al., 1984; Allan et al., 2001; Hertz et al., 2017; Mertins et al., 2017). Several other tick species have been detected in Florida but were not found in this study, likely due to host and environmental preferences and variation in abundance. For example, *Ixodes affinis* Neumann has been collected from other large mammals in the state, consistently from Florida panthers (*Puma concolor coryi* Bangs) and rarely from white-tailed deer, but to our knowledge has not been collected from wild pigs (Greiner et al., 1984; Wehinger et al., 1995; Allan et al., 2001; Hertz et al., 2017).

Hammock habitat produced the greatest number of all life-stages, particularly immatures, from drags. Hammock understories were covered in leaf litter with patchy vegetation, while both seminative and improved pastures were more likely to contain dense vegetation of varying heights, sometimes over 1.5 m. Increased vegetation height suspends the drag cloth above the ground, preventing contact with lower vegetation. This decreases the efficiency of dragging, particularly for immature stages which quest at lower heights than adults (Dobson et al., 2011). Additionally, the sheltered environment of hammocks reduces the saturation deficit, potentially allowing ticks to quest for longer periods of the year than in exposed habitats such as the seminative and improved pastures (Dobson et al., 2011), though we did not measure the saturation deficit in this study.

Three of the four tick species detected in this study are known vectors of human or animal pathogens. *Amblyomma maculatum* is the principal vector of both *Rickettsia parkeri* and *Hepatozoon americanum* in the southern United States, which cause *R. parkeri* rickettsiosis in humans and American canine hepatozoonosis in dogs, respectively (Teel et al., 2010; Parola et al., 2013). *Amblyomma maculatum* may also vector Panola Mountain Ehrlichia, which causes disease in humans and dogs (Loftis et al., 2016). Notably, *Amblyomma maculatum* is also a competent vector of *Ehrlichia ruminantium*, the causative agent of heartwater, a foreign disease of ruminants which can be devastating to naïve populations (Mahan et al., 2000; Allsopp, 2015). *Dermacentor variabilis* is historically considered the principal vector in the eastern United States of *Rickettsia rickettsii*, the causative agent of Rocky Mountain Spotted Fever in humans (Burgdorfer, 1975), and adults of this species are competent vectors of *Francisella tularensis*, the causative agent of tularemia in humans (Reese et al., 2011). *Dermacentor variabilis* is also a vector of *Anaplasma marginale*, the etiological agent of bovine anaplasmosis and currently the only tick-borne disease recognized to directly impact cattle production in the United States (Kocan et al., 2010a, b). However, for Florida strains of this pathogen, the transmission route is not well understood and may involve mechanical transmission rather than tick transmission (Kocan et al., 2004, 2010a, b). In the eastern United States, *Ixodes scapularis* is the principal vector of *Borrelia burgdorferi*, *Anaplasma phagocytophilum*, and deer tick virus (Powassan virus lineage II), the causative agents of Lyme disease, human anaplasmosis, and a viral encephalitis in humans, respectively (Nelder et al., 2016). However, deer tick virus has not yet been reported in Florida (Ebel, 2010; Hermance and Thangamani, 2017). Additionally, Florida has a low reported incidence of Lyme disease (Forrester et al., 2015), with the absence of *B. burgdorferi* detection in recently surveyed local populations of *I. scapularis* (Sayler et al., 2017). *Amblyomma auricularium* is an exotic species known to be established throughout southern Florida (Lord and Day, 2000; Mertins et al., 2017). *Amblyomma auricularium* is not known to be associated with disease; however, *Rickettsia* spp. of unknown pathogenicity have been detected in this species (Saraiva et al., 2013; Cohen et al., 2015; Lugarini et al., 2015).

In conclusion, our findings suggest that wild pigs are useful sentinel animals for the detection of adults of some tick species that are at low densities in the environment and for collection of greater numbers of adults of some ticks that are difficult to collect by dragging. Dragging was more productive than wild pig sampling for detecting immatures. We detected markedly higher numbers of *A. maculatum* adults by sampling wild pigs rather than by dragging. Both wild pig and drag surveys revealed comparable but low numbers of adult *A. auricularium* and *I. scapularis*, and wild pigs revealed a low number of *D. variabilis*, which drags did not detect. Our study focused on only the ears of pigs, and we likely missed detection of ticks due to this, although this still allowed for detection of adults of more tick species than dragging. However, our drag surveys were not targeted to specific times of day or microhabitats. Targeted host-seeking surveillance efforts or the use of additional techniques such as CO_2_ traps may have yielded greater numbers or possibly an increased richness of host-seeking ticks. Though it should not replace targeted sampling for species of particular interest, we found surveillance of wild pigs to be a useful starting point to understanding which tick species were present at this study site. There may be adults of additional ixodid tick species present at our study site which were not detected through either wild pig or drag surveys. As with all vector surveillance, the most appropriate method depends on the goals of the project.

When combined with ongoing wild pig research, hunting, or management programs, wild pig surveillance can provide a time- and cost-effective method for adult tick detection. Any wild pig surveillance program should incorporate proper health safety protective measures, as wild pigs host multiple diseases transmissible to humans and animals, including brucellosis and pseudorabies virus, respectively (Meng et al., 2009; Miller et al., 2017). Existing programs such as the United States Department of Agriculture's Animal and Plant Health Inspection Service National Feral Swine Damage Management Program can utilize wild pig sentinels as a long-term tick surveillance tool throughout the United States. These data would provide invaluable insight into the presence, distribution, and abundance of certain vectors of human, livestock, and wildlife disease.
