# Supplementary material for: Wild pigs as sentinels for hard ticks: A case study from south-central Florida
Source: Int J Parasitol Parasites Wildl. 2018 Apr 30;7(2):161–70. doi: 10.1016/j.ijppaw.2018.04.003 (PMC6032497; doi:10.1016/j.ijppaw.2018.04.003)
Supplement: Multimedia component 2 [file mmc2.doc]

Table S1. ANOVA table from the Poisson regression model for habitat and life stage associations with host-seeking tick density per ten square meters

| Term | Degrees of freedom | Test statistic (χ^2^) | P-value |
| --- | --- | --- | --- |
| Habitat type | 2 | 20.34 | 3.83e-05 |
| Life stage | 2 | 1660.12 | < 2e-16 |
| Habitat type:Life stage | 4 | 149.20 | < 2e-16 |

Table S2. Prevalence and 95% confidence intervals of tick infestation of wild pigs from May 22, 2015 to May 09, 2017 at Buck Island Ranch, Lake Placid, Florida

|  | Pigs sampled |  | Prevalence (95% CI) | | | |
| --- | --- | --- | --- | --- | --- | --- |
| 2015 |  |  | AMAU | AMMA | DEVA | IXSC |
| May | 7 |  | 0.0 (0.0, 0.0) | 42.9 (0.0, 83.3) | 28.6 (0.0, 66.7) | 0.0 (0.0, 0.0) |
| June | 10 |  | 0.0 (0.0, 0.0) | 20.0 (0.0, 50.0) | 20.0 (0.0, 50.0) | 0.0 (0.0, 0.0) |
| August | 14 |  | 0.0 (0.0, 0.0) | 78.6 (53.8, 100.0) | 7.1 (0.0, 25.0) | 0.0 (0.0, 0.0) |
| December | 34 |  | 0.0 (0.0, 0.0) | 58.8 (41.7, 74.3) | 5.9 (0.0, 15.0) | 11.8 (2.6, 23.3) |
| 2016 |  |  |  |  |  |  |
| January | 11 |  | 0.0 (0.0, 0.0) | 54.5 (23.1, 85.7) | 9.1 (0.0, 28.6) | 9.1 (0.0, 30.0) |
| February | 4 |  | 0.0 (0.0, 0.0) | 0.0 (0.0, 0.0) | 0.0 (0.0, 0.0) | 0.0 (0.0, 0.0) |
| March | 26 |  | 0.0 (0.0, 0.0) | 3.8 (0.0, 12.9) | 0.0 (0.0, 0.0) | 0.0 (0.0, 0.0) |
| August | 2 |  | 0.0 (0.0, 0.0) | 50.0 (0.0, 100.0) | 0.0 (0.0, 0.0) | 0.0 (0.0, 0.0) |
| September | 26 |  | 0.0 (0.0, 0.0) | 96.2 (86.7, 100.0) | 0.0 (0.0, 0.0) | 0.0 (0.0, 0.0) |
| October | 81 |  | 7.4 (2.4, 13.6) | 70.4 (60.3, 80.2) | 1.2 (0.0, 4.1) | 1.2 (0.0, 4.1) |
| November | 1 |  | 0.0 (0.0, 0.0) | 100.0 (100.0, 100.0) | 0.0 (0.0, 0.0) | 0.0 (0.0, 0.0) |
| 2017 |  |  |  |  |  |  |
| February | 7 |  | 0.0 (0.0, 0.0) | 0.0 (0.0, 0.0) | 0.0 (0.0, 0.0) | 0.0 (0.0, 0.0) |
| April | 85 |  | 4.7 (1.1, 9.9) | 0.0 (0.0, 0.0) | 1.2 (0.0, 3.8) | 0.0 (0.0, 0.0) |
| May | 8 |  | 0.0 (0.0, 0.0) | 0.0 (0.0, 0.0) | 0.0 (0.0, 0.0) | 0.0 (0.0, 0.0) |
| **Total Study** | **316** |  | **3.2 (1.3, 5.2)** | **40.2 (35.1, 45.8)** | **3.2 (1.4, 5.3)** | **1.9 (0.6, 3.6)** |
| Calculations based on adults identified to species. Prevalence calculated as the number of pigs infested divided by the number of pigs surveyed and expressed as a percentage. AMAU = *A. auricularium*, AMMA = *A. maculatum*, DEVA = *D. variabilis*, IXSC = *I. scapularis* | | | | | | |

Table S3. Abundance and 95% confidence intervals of tick infestation of wild pigs from May 22, 2015 to May 09, 2017 at Buck Island Ranch, Lake Placid, Florida

|  | Pigs sampled |  | Abundance (95% CI) | | | |
| --- | --- | --- | --- | --- | --- | --- |
| 2015 |  |  | AMAU | AMMA | DEVA | IXSC |
| May | 7 |  | 0.00 (0.00, 0.00) | 1.57 (0.00, 4.00) | 0.43 (0.00, 1.00) | 0.00 (0.00, 0.00) |
| June | 10 |  | 0.00 (0.00, 0.00) | 0.40 (0.00, 1.11) | 0.20 (0.00, 0.50) | 0.00 (0.00, 0.00) |
| August | 14 |  | 0.00 (0.00, 0.00) | 10.57 (3.84, 17.75) | 0.07 (0.00, 0.23) | 0.00 (0.00, 0.00) |
| December | 34 |  | 0.00 (0.00, 0.00) | 2.15 (1.22, 3.34) | 0.06 (0.00, 0.15) | 0.18 (0.03, 0.39) |
| 2016 |  |  |  |  |  |  |
| January | 11 |  | 0.00 (0.00, 0.00) | 1.36 (0.47, 2.40) | 0.18 (0.00, 0.60) | 0.09 (0.00, 0.30) |
| February | 4 |  | 0.00 (0.00, 0.00) | 0.00 (0.00, 0.00) | 0.00 (0.00, 0.00) | 0.00 (0.00, 0.00) |
| March | 26 |  | 0.00 (0.00, 0.00) | 0.04 (0.00, 0.12) | 0.00 (0.00, 0.00) | 0.00 (0.00, 0.00) |
| August | 2 |  | 0.00 (0.00, 0.00) | 1.50 (0.00, 3.00) | 0.00 (0.00, 0.00) | 0.00 (0.00, 0.00) |
| September | 26 |  | 0.00 (0.00, 0.00) | 9.65 (6.77, 12.74) | 0.00 (0.00, 0.00) | 0.00 (0.00, 0.00) |
| October | 81 |  | 0.09 (0.02, 0.17) | 5.74 (4.17, 7.43) | 0.01 (0.00, 0.04) | 0.01 (0.00, 0.04) |
| November | 1 |  | 0.00 (0.00, 0.00) | 8.00 (8.00, 8.00) | 0.00 (0.00, 0.00) | 0.00 (0.00, 0.00) |
| 2017 |  |  |  |  |  |  |
| February | 7 |  | 0.00 (0.00, 0.00) | 0.00 (0.00, 0.00) | 0.00 (0.00, 0.00) | 0.00 (0.00, 0.00) |
| April | 85 |  | 0.05 (0.01, 0.10) | 0.00 (0.00, 0.00) | 0.01 (0.00, 0.04) | 0.00 (0.00, 0.00) |
| May | 8 |  | 0.00 (0.00, 0.00) | 0.00 (0.00, 0.00) | 0.00 (0.00, 0.00) | 0.00 (0.00, 0.00) |
| **Total Study** | **316** |  | **0.03 (0.01, 0.06)** | **3.10 (2.44, 3.82)** | **0.04 (0.02, 0.06)** | **0.03 (0.01, 0.05)** |
| Calculations based on adults identified to species. Abundance calculated as the sum of ticks collected divided by the number of pigs surveyed. AMAU = *A. auricularium*, AMMA = *A. maculatum*, DEVA = *D. variabilis*, IXSC = *I. scapularis* | | | | | | |

Table S4. Intensity and 95% confidence intervals of tick infestation of wild pigs from May 22, 2015 to May 09, 2017 at Buck Island Ranch, Lake Placid, Florida

|  | Pigs sampled |  | Intensity (95% CI) | | | |
| --- | --- | --- | --- | --- | --- | --- |
| 2015 |  |  | AMAU | AMMA | DEVA | IXSC |
| May | 7 |  | - | 3.67 (1.00, 8.00) | 1.50 (1.00, 2.00) | - |
| June | 10 |  | - | 2.00 (1.00, 3.00) | 1.00 (1.00, 1.00) | - |
| August | 14 |  | - | 13.45 (5.83, 22.17) | 1.00 (1.00, 1.00) | - |
| December | 34 |  | - | 3.65 (2.33, 5.36) | 1.00 (1.00, 1.00) | 1.50 (1.00, 3.00) |
| 2016 |  |  |  |  |  |  |
| January | 11 |  | - | 2.50 (1.50, 3.67) | 2.00 (2.00, 2.00) | 1.00 (1.00, 1.00) |
| February | 4 |  | - | - | - | - |
| March | 26 |  | - | 1.00 (1.00, 1.00) | - | - |
| August | 2 |  | - | 3.00 (3.00, 3.00) | - | - |
| September | 26 |  | - | 10.04 (6.96, 13.33) | - | - |
| October | 81 |  | 1.17 (1.00, 1.60) | 8.16 (6.26, 10.33) | 1.00 (1.00, 1.00) | 1.00 (1.00, 1.00) |
| November | 1 |  | - | 8.00 (8.00, 8.00) | - | - |
| 2017 |  |  |  |  |  |  |
| February | 7 |  | - | - | - | - |
| April | 85 |  | 1.00 (1.00, 1.00) | - | 1.00 (1.00, 1.00) | - |
| May | 8 |  | - | - | - | - |
| **Total Study** | **316** |  | **1.10 (1.00, 1.33)** | **7.71 (6.35, 9.16)** | **1.20 (1.00, 1.50)** | **1.33 (1.00, 2.20)** |
| Calculations based on adults identified to species. Intensity calculated as the sum of ticks collected divided by the number of pigs infested. AMAU = *A. auricularium*, AMMA = *A. maculatum*, DEVA = *D. variabilis*, IXSC = *I. scapularis* | | | | | | |

Table S5. Estimated mean densities of host-seeking ticks per 10m^2^ by life stage and habitat type with 95% confidence intervals (CI)

| Habitat | Life stage | Estimated mean density (95% CI) |
| --- | --- | --- |
| Hammock | Larva | 1.53 (1.47, 1.58) |
|  | Nymph | 7.54e-02 (6.38e-02, 8.91e-02) |
|  | Adult | 1.15e-02 (7.48e-03, 1.76e-02) |
| Improved | Larva | 3.25e-04 (4.58e-05, 2.31e-03) |
|  | Nymph | 2.60e-03 (1.30e-03, 5.20e-03) |
|  | Adult | 1.30e-03 (4.88e-04, 3.47e-03) |
| Seminative | Larva | 3.73e-03 (2.17e-03, 6.42e-03) |
|  | Nymph | 1.15e-03 (4.31e-04, 3.06e-03) |
|  | Adult | 4.02e-03 (2.38e-03, 6.78e-03) |
